# Case Report: resolution of refractory seizures after neurosurgical intervention in newborns with cerebral extra-axial hemorrhages

**DOI:** 10.3389/fped.2024.1388454

**Published:** 2024-08-16

**Authors:** C. Rohaert, J. K. H. Spoor, M. Dremmen, A. M. van Hengel-Jacobs, L. S. Smit, R. Knol

**Affiliations:** ^1^ Division of Neonatology, Department of Neonatal and Pediatric Intensive Care, Erasmus MC—Sophia Children’s Hospital, Rotterdam, Netherlands; ^2^ Department of Neurosurgery, Erasmus MC—Sophia Children’s Hospital, Rotterdam, Netherlands; ^3^ Division of Pediatric Radiology, Department of Radiology and Nuclear Medicine, Erasmus MC—Sophia Children’s Hospital, Rotterdam, Netherlands; ^4^ Department of Pediatrics, Franciscus Gasthuis & Vlietland Hospital, Rotterdam, Netherlands; ^5^ Division of Neurology, Department of Pediatric Neurology, Erasmus MC—Sophia Children’s Hospital, Rotterdam, Netherlands

**Keywords:** intracranial hemorrhage, extra-axial hemorrhage, neonate, refractory seizures, neurosurgical decompression, needle aspiration, case report

## Abstract

**Introduction:**

Intracranial hemorrhage is a significant cause of neurological damage in newborns. Extra-axial hemorrhages with intraparenchymal extension can precipitate acute clinical deterioration. Seizures are one of the presenting symptoms, which can be refractory to treatment. These hemorrhages can result in considerable long-term morbidity and mortality.

**Aim:**

The objective of this report was to present three cases of extra-axial hemorrhages in neonates, all exhibiting refractory seizures that resolved after neurosurgical intervention. In addition, a review of literature is provided.

**Methods:**

Data collected included clinical history, laboratory findings, neuroimaging studies, type of neurosurgical intervention, and patient outcome. All infants presented with extra-axial hemorrhages along with clinical and radiological signs of increased intracranial pressure within the first 6 days of life. These manifestations included a decreased level of consciousness, hypertension, bradycardia, and cerebral midline shift on imaging. Refractory seizures were present in all cases. Urgent magnetic resonance imaging was performed followed by neurosurgical intervention (two needle aspirations, one cranial trepanation), leading to amelioration of clinical symptoms and complete resolution of seizures. Follow-up outcomes included normal psychomotor development in one infant, mild cerebral paresis in another, and delayed motor development in the third. None of the infants developed epilepsy.

**Conclusion:**

This study underscores the critical importance of monitoring seizure activity, conducting urgent and appropriate imaging, and implementing targeted neurosurgical intervention, preferably through minimally invasive methods such as percutaneous needle aspiration. Clinicians should be aware of this clinical picture and respond promptly to mitigate neurological damage.

## 1 Introduction

Intracranial hemorrhage (ICH) is defined as the pathological accumulation of blood within the cranial cavity and may have devastating consequences on morbidity and mortality (1). ICH in preterm infants is common and typically presents as intraventricular bleeding (1, 2). In contrast, ICH in term infants is less common and differs in location, etiology, clinical presentation, and outcome. Term infants primarily present with an extra-axial hemorrhage, particularly subdural hemorrhages, or less frequently, intra-axial intraparenchymal hemorrhages (1, 3). Subdural, epidural, and subarachnoid hemorrhages are extra-axial hemorrhages that can occur both supratentorially and infratentorially (1, 4).

The outcome of extra-axial hemorrhages strongly depends on the type, location, and extent of the hemorrhage. Of neonates with a posterior fossa subdural hemorrhage, 80%–88% have a good outcome; of those with moderate to large convexity subdural hemorrhages, 50%–90% have good follow-up outcomes. Deficits are mostly related to the presence of parenchymal extension. The more extensive the bleeding, the worse the outcome, with a possibly fatal course and major sequalae. Neonates with primary subarachnoid hemorrhages generally have a good outcome, especially when they are minimally symptomatic. At least 90% of full-term infants with seizures as the primary manifestation are normal at follow-up (4). However, since ICH often occurs at multiple sites, predicting the prognosis is challenging.

In term infants, ICH may be present without manifestation of clinical symptoms, so the true incidence is not precisely known (1, 3).

Not all term infants with ICH present with clinical symptoms, so the real incidence is not exactly known (1, 3). One study detected ICH in 9% of 725 term and late-preterm infants who underwent postnatal magnetic resonance imaging (MRI) of the brain (5). In 8% of asymptomatic term infants, subdural hemorrhage was detected on brain MRI, with complete resolution by 4 weeks of age (6). Looney et al. reported an even higher prevalence of asymptomatic ICH in 26% of term neonates after vaginal birth (7).

ICH can produce neurological symptoms within the first few days of life, including increasing head circumference, bulging fontanelle, apneas, altered level of consciousness, seizures, and bradycardia (2, 8). When a newborn presents with seizures, symptomatic anti-epileptic treatment must be administered promptly, followed by investigations to determine the underlying cause. In particular, if seizures become refractory, failing to respond to two anti-epileptic drugs (9), urgent diagnostics and interventions are required (8). The aim of the present case series was to underscore the importance of accurate and rapid imaging in neonates presenting with therapy-refractory seizures and to advocate for urgent neurosurgical intervention to resolve seizures by reducing cortical irritation, thus preventing further neurological deterioration.

## 2 Case reports

### 2.1 Case 1

**Figure 1:**
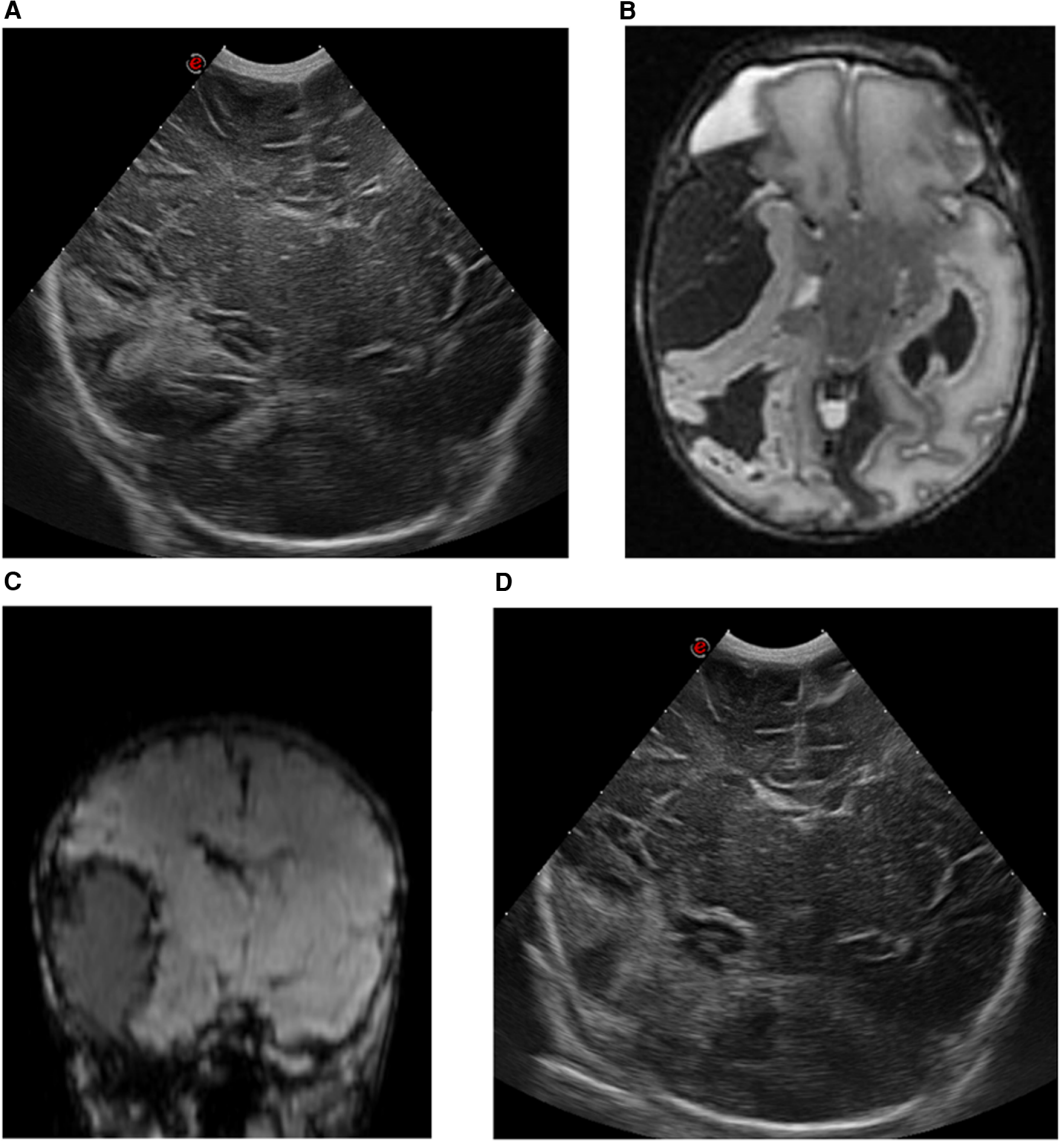
cUS and MRI findings of case 1. **(A)** cUS coronal view, showing a right-sided echogenic lesion temporo-occipital with midline shift to the left. **(B)** MRI T2-weighted sequence, axial view, and **(C)** MRI susceptibility-weighted imaging sequence, coronal view, showing an intra-axial hemorrhage in the right temporo-occipital region with an extra-axial component in the frontotemporal region, with a maximal thickness of 32 mm. This causes a mass effect on the right temporal lobe with midline shift of 5 mm to the left. **(D)** cUS coronal view, after surgical intervention.

A female infant weighing 3,550 g (P50) was born at 40^4//7^ weeks to a primigravida mother who had received routine prenatal care. There was no relevant parental history. The infant was born via non-assisted vaginal delivery. The APGAR scores were 7 and 8 at 1 and 5 min, respectively. There was prolonged rupture of membranes (48 h), maternal fever, and meconium-stained amniotic fluid. The umbilical cord blood gas showed a pH of 7.15 and the initial physical examination was normal. The patient was admitted to the neonatal ward for respiratory support through continuous positive airway pressure due to transitional issues or suspected infection. At 4 h postpartum (day 0), the infant exhibited somnolence and suspected seizures characterized by central apneas, gazing, and nystagmus. A loading dose of phenobarbital (20 mg/kg) was administered. Due to persistent central apneas, the infant was intubated and transferred to our tertiary-level neonatal intensive care unit (NICU) for mechanical ventilation and neuromonitoring. Cranial ultrasound (cUS) revealed a large right-sided echogenic temporo-occipital lesion with a mild midline shift to the left (Figure 1A). The complete blood count (CBC) showed a normal hemoglobin level and platelet count. Other laboratory tests, including coagulation profile and metabolic panel, were within normal ranges. Family history revealed no clotting problems. Amplitude integrated electroencephalography (aEEG) indicated seizure activity in the right hemisphere. Refractory seizures developed from day 0 to 1, necessitating the administration of midazolam (loading dose: 0.1 mg/kg), lidocaine (loading dose: 2 mg/kg, followed by continuous infusion for 30 h, then tapered gradually), midazolam infusion (up to 300 µg/kg/h) and lacosamide (1 mg/kg/dose). An urgent brain MRI examination confirmed an extensive extra-axial hemorrhage in the right frontotemporal region with mass effect on the right frontal and temporal lobes, and midline shift to the left. There was also intra-axial blood in the temporo-occipital region and an intraventricular hemorrhage on the right side, with a more limited extra-axial hemorrhage in the left temporal region (Figures 1B,C). Due to refractory seizures and imminent brain herniation presenting with bradycardia and hypertension, a neurosurgical intervention was performed by needle aspiration of 20 ml of blood. Post-procedure aEEG showed complete cessation of seizure activity, allowing for the tapering of anti-epileptic drugs. Serial cUS over the following days showed a further decrease in the size of the hemorrhage and reduction of the midline shift, without signs of rebleeding (Figure 1D).

**Figure 2:**
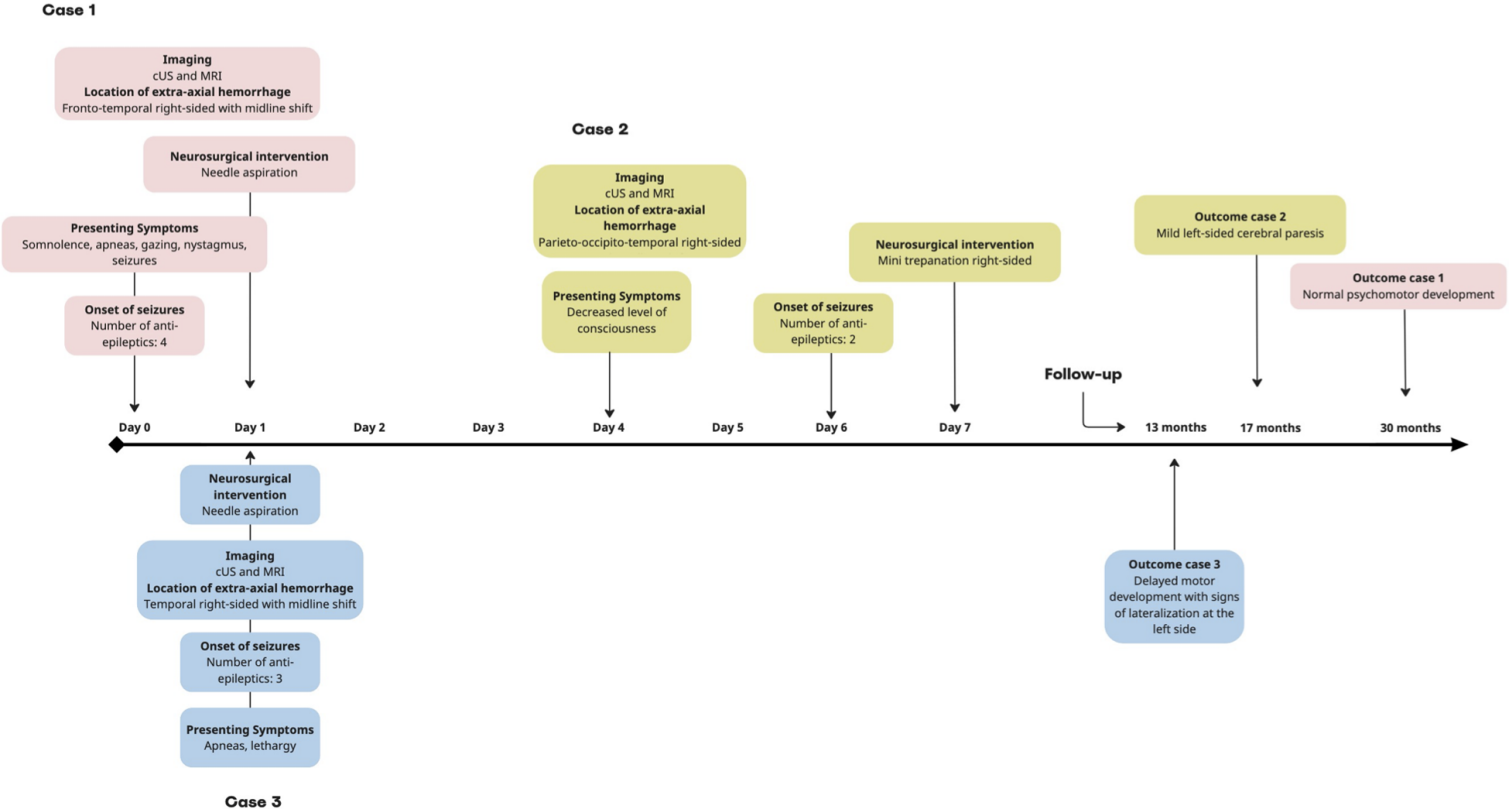
Timeline of the neurological course of cases 1–3. In all cases, the seizures ceased after neurosurgical intervention. cUS, cerebral ultrasound; MRI, magnetic resonance imaging.

The follow-up at 6 months revealed delayed gross motor development due to severe hyperlaxity with a normal score at the Alberta Infant Motor Scale (AIMS) diagnosed by a pediatric neurologist and physiotherapist with standardized neurological exam. At 30 months, psychomotor development was normal according to the Bayley Scales of Infant and Toddler Development, Third Edition (BSID III). An overview of this case is shown in Figure 2.

### 2.2 Case 2

**Figure 3:**
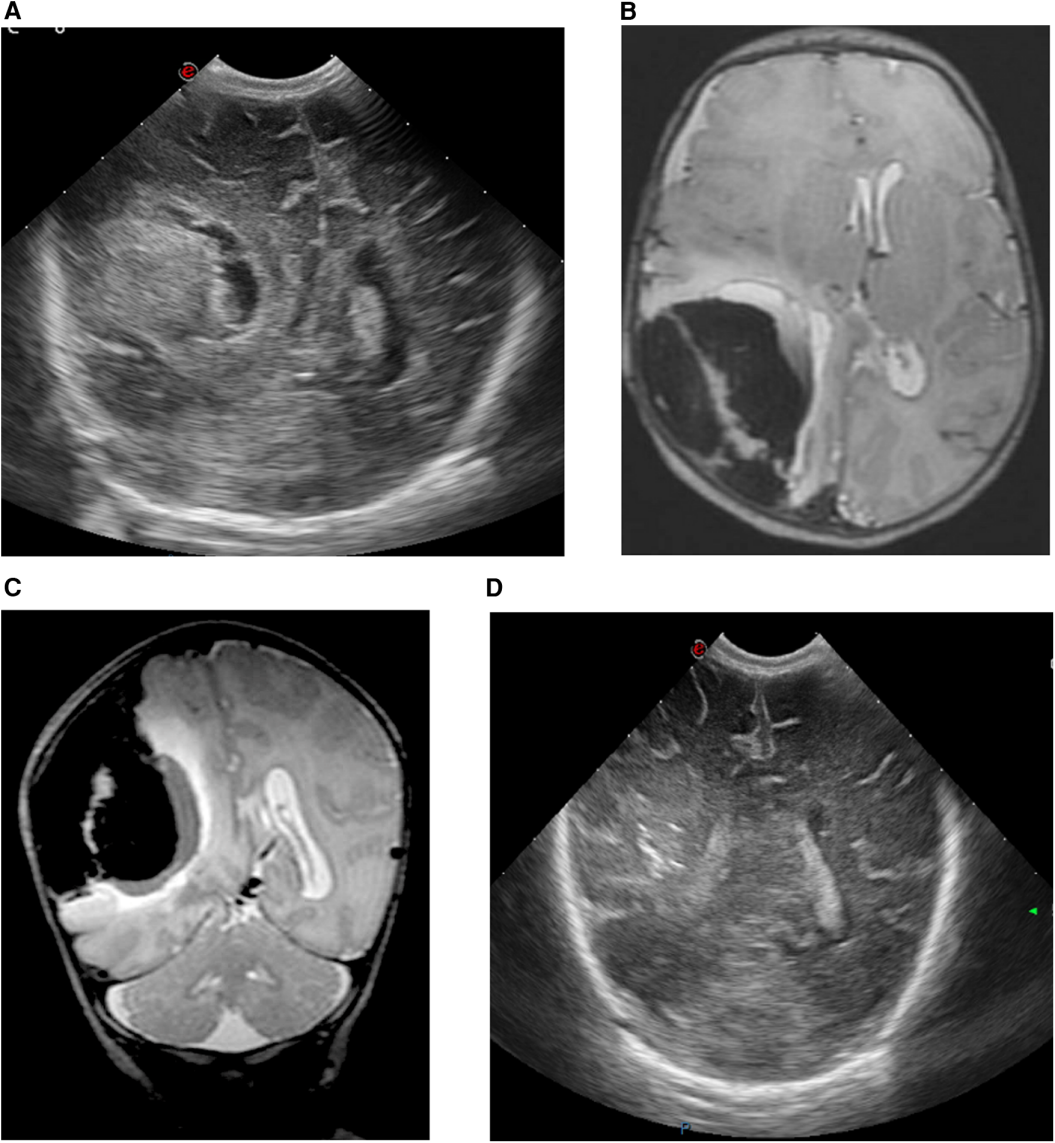
cUS and MRI brain findings of case 2. **(A)** cUS coronal view, showing a large echogenic parieto-occipital lesion at the right side, presumably extra-axial hemorrhage with midline shift to the left side. **(B)** MRI T2-weighted sequence, axial view, and **(C)** MRI T2-weighted sequence, coronal view, showing a large extra-axial hemorrhage in the parieto-occipito-temporal region on the right side with a maximal thickness of 43 mm and a midline shift of 8 mm to the left. **(D)** cUS coronal view, after craniotomy showing a decrease in the size of the hematoma, reduction of the midline shift and slender ventricles.

A female infant weighing 2,550 g (P20) was born to a gravida 2, para 1 mother at 37^1/7^ weeks who had received routine prenatal care with no remarkable parental history. The infant was born via non-assisted vaginal delivery. The APGAR scores were 9 and 10 at 1 and 5 min, respectively. The umbilical cord blood gas and physical examination were normal. The infant was discharged at day 1. On day 4, the infant was readmitted due to inconsolable crying, hypotonia, and feeding difficulties. By the end of day 6, repetitive clonic movements on the left side were observed, prompting transfer to a tertiary-level NICU for neuromonitoring. cUS revealed a large echogenic right-sided parieto-occipital lesion, presumed to be extra-axial, with a midline shift to the left (Figure 3A). The CBC showed mild anemia and a normal platelet count. Other laboratory tests, including coagulation profile and metabolic panel, were within normal ranges. aEEG indicated seizure activity in the right hemisphere. A loading dose of phenobarbital (20 mg/kg) was administered, with seizure recurrence necessitating a repeat dose of 10 mg/kg. On day 7, there was recurrence of seizures, necessitating the administration of lidocaine (loading dose: 2 mg/kg, followed by continuous infusion for 30 h, then tapered gradually). The child was intubated and mechanically ventilated due to a decreased level of consciousness. An urgent brain MRI confirmed the cUS findings of a large extra-axial hemorrhage in the right parieto-occipito-temporal region, with midline shift and uncal herniation (Figures 3B,C). Neurosurgical intervention was performed, consisting of a small trepanation of the right skull with aspiration of 40 ml of blood. Post-procedure aEEG showed complete cessation of seizure activity, allowing for the tapering of anti-epileptic drugs. Serial cUS over the following days showed a decrease in the size of the hematoma without rebleeding and reduction of the midline shift, with normal ventricles (Figure 3D). A brain MRI scan at 2 months showed further reduction of the extra-axial hemorrhage. There was tissue loss in the right parieto-occipital region with *ex vacuo* dilatation at the dorsal part of the ventricle. No arteriovenous malformation was detected on magnetic resonance angiography (MRA). The infant developed left-sided cerebral palsy, which was still mildly present at 17 months of age, clinically diagnosed according to the Surveillance of Cerebral Palsy in Europe network by a pediatric neurologist with a standardized neurological exam. In addition, esotropia developed at 17 months of age. An overview of case 2 is shown in Figure 2.

### 2.3 Case 3

A female infant weighing 1,420 g (*P* < 3) was born to a primigravida mother at 33^3/7^ weeks.

**Figure 4:**
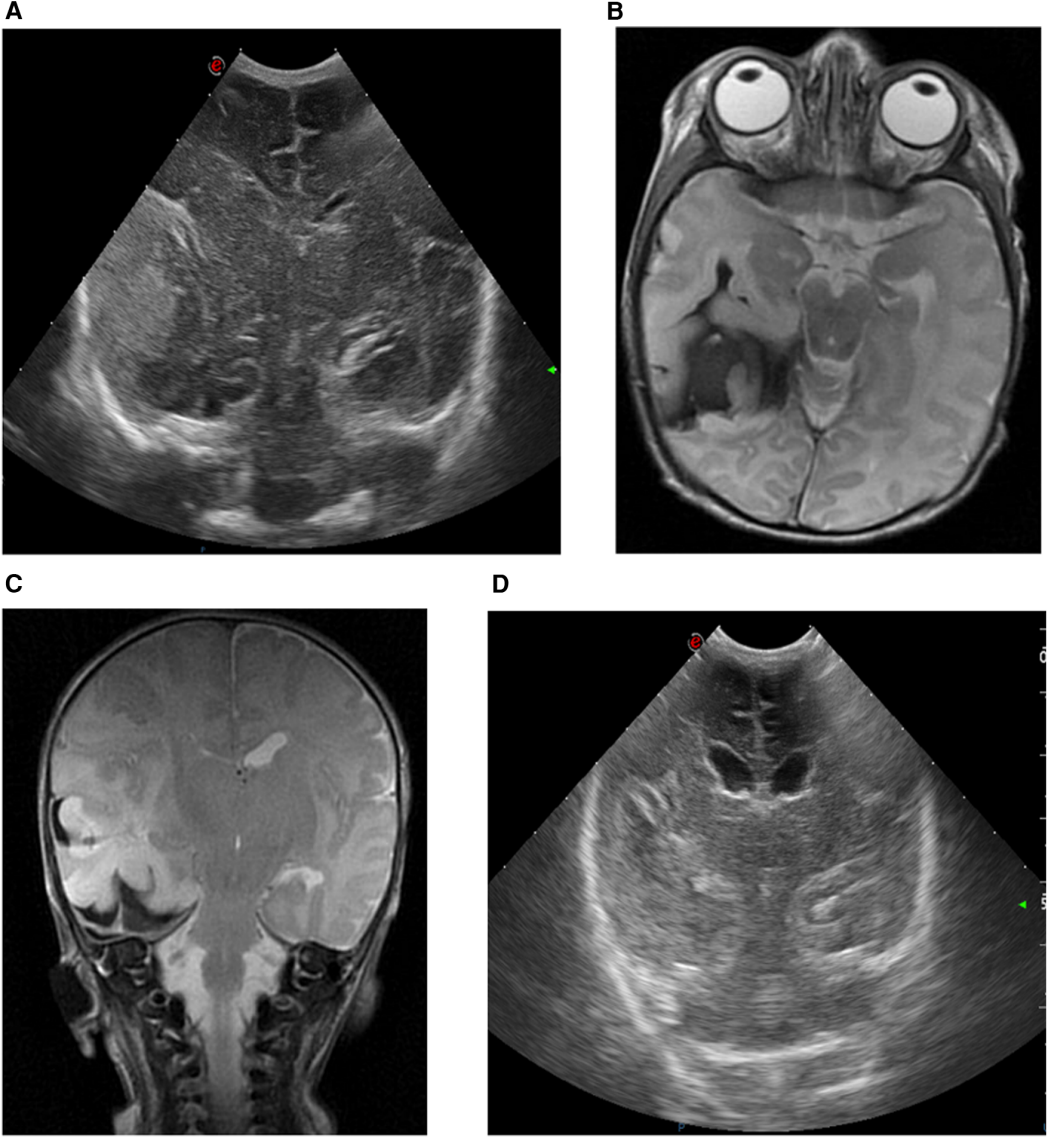
cUS and MRI brain findings of case 3. **(A)** cUS coronal view, showing a right-sided echogenic extra-axial lesion, presumably subdural and temporally located, with midline shift. **(B)** MRI T2-weighted sequence, axial view, and **(C)** MRI T2-weighted sequence, coronal view, showing a large extra-axial hemorrhage in the temporal region on the right side with a maximal thickness of 16 mm and a midline shift of 7 mm to the left. The combined diameter of the subdural hematoma and intraparenchymal hemorrhage is 29 mm. **(D)** cUS coronal view, after surgical intervention.

Parental history was unremarkable. The infant presented with intrauterine growth restriction and abnormal umbilical artery Doppler waveforms were suggestive of placental insufficiency. Steroids were administered to the mother due to imminent premature delivery and the infant was born via caesarian section due to decelerative cardiotocography. The APGAR scores were 9 and 9 at 1 and 5 min, respectively. Umbilical cord blood gas analysis was normal, but multiple diffuse cutaneous hematomas were noted upon clinical examination. At 8 h of age, the infant exhibited recurrent apneic episodes leading to respiratory insufficiency and became progressively more lethargic. After intubation, she was transferred to a tertiary-level NICU. cUS revealed a large right-sided echogenic extra-axial temporal lesion, likely subdural, causing a midline shift (Figure 4A). CBC showed mild anemia and severe thrombocytopenia. The coagulation profile indicated a prolonged prothrombin time. Transfusions of erythrocyte, platelets, and plasma were administered, alongside vitamin K supplementation. aEEG indicated subclinical seizures originating from the right hemisphere. A loading dose of levetiracetam (20 mg/kg) was administered with recurrence of seizures, prompting a second dose of 20 mg/kg. Midazolam was added (loading dose: 0.05 mg/kg, followed by continuous infusion), followed by lidocaine (loading dose: 2 mg/kg, followed by continuous infusion for 22 h, then tapered gradually) due to refractory seizures. Urgent brain MRI confirmed cUS findings, revealing a large extra-axial temporal hemorrhage at the right side with a midline shift to the left. In addition, there was right-sided subdural blood at the tentorium, as well as interhemispheric and intraventricular hemorrhage (Figures 4B,C). The infant exhibited a decreased level of consciousness upon clinical examination. Because of the radiological signs of a transtentorial herniation, midline shift, and ongoing seizures, the neurosurgeon performed a needle aspiration with the evacuation of 15 ml of blood. Post-procedure aEEG demonstrated complete cessation of the seizure activity and subsequently the anti-epileptic therapy was tapered. Serial cUS showed a reduction in hematoma size without rebleeding and resolution of the midline shift (Figure 4D). Thrombocytopenia, attributed to dysmaturity and consumption secondary to intracerebral hemorrhage, normalized spontaneously. The TORCH panel was negative and fetal/neonatal allo-immune thrombocytopenia was excluded. A follow-up brain MRI examination at 1 week after the procedure showed a further reduction in the temporal hematoma without parenchymal ischemia. Partial compensatory dilatation of the occipital horn of the right lateral ventricle was noted. Magnetic resonance venography (MRV) showed normal findings. There was a normal cognitive development but delayed motor skills as determined by the AIMS and standardized neurological exam. There was a preference for the left hand without difference in tone or posture at 13 months of age, diagnosed by a pediatric neurologist and physiotherapist. In addition, the infant developed infantile esotropia. The patient was lost to follow-up. An overview of case 3 is shown in Figure 2.

## 3 Discussion

ICH represents a significant morbidity in the neonatal population, posing considerable risks for impaired neurodevelopment (10, 11). In this case series, all infants presented with severe symptoms attributable to extra-axial hemorrhages, leading to secondary compression and midline shift. Urgent brain MRI was performed when refractory seizures persisted, followed by neurosurgical intervention. Neuroimaging is essential for differentiating the location and extent of bleeding and assessing the impact on the brain. The degree of brain involvement, along with the presence and duration of seizures, has impact on outcome in newborn infants (10, 12). cUS is an effective screening tool for suspected ICH due to its ease of use, portability, and absence of radiation (1). It promptly guides clinicians in the context of a broader differential diagnosis. However, if refractory seizures persist, additional neuroimaging is warranted, as cUS is less detailed and may fail to visualize important information (13). Visualization of an extra-axial hemorrhage by cUS can be challenging (12). Brain MRI is superior, offering higher sensitivity in detecting extra-axial hemorrhages (2). When quickly available, brain MRI is preferable to computed tomography due to the absence of radiation and the provision of more detailed imaging, particularly concerning parenchymal involvement and associated ischemic changes in the brain (13).

The types of ICH in relation to seizures have not been thoroughly studied (11). Involvement of multiple compartments is possible (10), as was observed in our cases. Multisite ICH is associated with a higher incidence of subclinical seizures and are more challenging to manage with medication compared to single ICH (11). Extra-axial hemorrhages may result from the rupture of veins in the subdural space, leading to bleeding from the venous sinus (3). Subarachnoid hemorrhages may cause seizures based on direct contact with neurons. Subdural hemorrhages present more often asymptomatic in neonates (11, 13), but rapid clinical deterioration due to brainstem compression has also been reported (1, 13–15). Subdural hemorrhage in the anterior and middle fossae, often associated with subarachnoid hemorrhage, can result in seizures (13). Neonatal epidural hemorrhage is a well-documented complication of forceps- and vacuum-assisted deliveries (13, 16–19). Seizures in the presence of these hemorrhages are usually due to other brain injury (13, 16). The most common clinical seizure type in extra-axial hemorrhages tends to be focal or subtle (4). Intraparenchymal hemorrhages are less frequently reported (1), but those involving cortical and subcortical gray matter can cause seizures (11, 13). Hemorrhage in the temporal lobe can manifest with apneic seizures (1, 14), as observed in cases 1 and 3.

Neonates with refractory seizures are at high risk of severe neurological disability and development of epilepsy (11). However, distinguishing whether the damage is attributed to seizures themselves or the underlying brain lesions causing them remains challenging (20). Prolonged seizure activity tends to become more refractory over time, necessitating additional medications with associated morbidity (21). Thus, performing aEEG in cases of ICH is recommended for the detection of subclinical seizures (1, 11).

Birth trauma, prolonged labor, and instrumental delivery are well described risk factors for ICH in term infants (1, 2). In the majority of neonates, ICH occurs spontaneously. Sometimes it is due to a clotting defect such as allo- or iso-immune thrombocytopenia or inherited coagulation disorders. A CBC and baseline coagulation profile are recommended initially (22). Thrombocytopenia was observed in one of our cases; allo-immune thrombocytopenia and perinatal infection were ruled out. It was attributed to pre- and dysmaturity. Coagulation profiles showed no abnormalities in our cases. Children with a bleeding disorder, acquired or inherited, tend to have more severe ICH and consequences (1). Imaging of the vessels using brain MRA or cerebral angiography is often performed to exclude vascular malformations. These malformations are frequently not detectable on imaging in the acute phase of the hemorrhage, as the bleeding can compress and obscure them. When the etiology is unclear, delayed imaging is useful after the bleeding has resolved (23). This is especially true for children aged 2 years or older, as Boulouis et al. (24) stated that vascular lesions are the most frequent cause of ICH in this age group, whereas coagulation disorders and cardiac diseases accompanied by anticoagulation therapy are more common causes of ICH in children aged under 2 years. Vein of Galen aneurysmal malformation, dual sinus malformation, and pial arteriovenous fistula are the most common vascular malformations in neonates; however, they rarely present with neonatal hemorrhage. Vein of Galen aneurysmal malformation and dual sinus malformation can mimic a subdural hemorrhage (25). In our case series, we performed MRV 1 week after the intervention in case 3, which showed normal results. We did not repeat the MRV later because we had already identified a reliable cause for ICH. In the second case, we did perform delayed vascular imaging since there was no explanation for the ICH. In case 1, MRA was not performed due to the presence of primarily extra-cerebral blood in the context of a traumatic birth and the consequently low suspicion of vascular malformation.

Neurosurgical intervention is beneficial for the quick resolution of symptoms associated with ICH. Indications for neurosurgical intervention include clinical symptoms of increased intracranial pressure (such as Cushing's triad, decreased consciousness), refractory seizures, and radiological evidence of mass effect (26). Cizmeci et al. (10) reported eight neonates with intra- and extra-axial cerebral hemorrhages resulting in signs of increased intracranial pressure and midline shift. All presented with refractory seizures and were treated with bedside ultrasound-guided needle puncture, leading to immediate symptom improvement. A similar amelioration of seizure activity was observed in our cases.

Vinchon et al. (27) conducted a prospective study on traumatic intracranial hemorrhage in neonates. They stated that the decision for decompression depends on the clinical tolerance of the injury and the extent of the hemorrhage. These authors discuss the role of needle aspiration, which is a less invasive procedure and, if successful, may obviate the need for surgical decompression. Craniotomy may be excessively invasive for already unstable neonates (16). The invasive nature of craniotomy increases the risk of complications, such as bleeding and exacerbation of hemodynamic instability, along with the necessity for general anesthesia (10, 16). Needle aspiration is considered a less invasive treatment option for extra-axial hemorrhages or hematomas approaching the surface. Several authors (16–18, 28) have reported success with needle aspiration in extra-axial hemorrhages. Supporting the use of needle aspiration, Noguchi et al. (16) noted that a neonatal epidural hemorrhage tends to be more liquefied compared to adults, facilitating needle aspiration. In addition, Cizmeci et al. (10) reported favorable outcomes with needle aspiration. Ultrasound guidance can minimize the risk of brain or vascular injury and can be performed bedside. In their cases, symptoms manifested early (on day 1 or 2), which may explain the success of needle aspiration since the blood is more fluid in the hyperacute phase. Needle aspiration was also used in our cases 1 and 3, as we anticipated that the aspirated blood would be fluid due to the early onset of symptoms. Using ultrasound guidance, the needle is inserted via a suture or fontanel. The procedure takes place bedside or in the operation room when the chance of having to convert to a craniotomy is deemed high. Case 2 presented with later-onset symptoms and deterioration on day 7. In this case, craniotomy was chosen as the blood would likely have clotted, making needle aspiration unsuccessful.

## 4 Conclusion

Based on the reported cases and reviewed literature, we recommend immediate imaging using cUS for all newborn infants presenting with neurological symptoms. In the presence of an ICH, early initiation of aEEG to detect seizures is advised, with prompt treatment being mandatory. If seizures are refractory, further imaging with brain MRI and subsequent neurosurgical intervention is recommended. Timely multidisciplinary management is essential and improves the clinical decision-making process. A comprehensive diagnostic evaluation for underlying pathology is necessary. We speculate that the relatively favorable outcomes in these cases are attributable to timely diagnosis and intervention. Despite prompt and adequate treatment, the risk of delayed neurodevelopmental outcome persists.

## Data Availability

The original contributions presented in the study are included in the article/Supplementary Material, further inquiries can be directed to the corresponding author.
